# A Reduction in Video Gaming Time Produced a Decrease in Brain Activity

**DOI:** 10.3389/fnhum.2019.00134

**Published:** 2019-04-17

**Authors:** Diankun Gong, Yutong Yao, Xianyang Gan, Yurui Peng, Weiyi Ma, Dezhong Yao

**Affiliations:** ^1^The Clinical Hospital of Chengdu Brain Science Institute, MOE Key Lab for Neuroinformation, University of Electronic Science and Technology of China, Chengdu, China; ^2^Center for Information in Medicine, School of Life Science and Technology, University of Electronic Science and Technology of China, Chengdu, China; ^3^Faculty of Natural Science, University of Stirling, Stirling, United Kingdom; ^4^School of Human Environmental Sciences, University of Arkansas, Fayetteville, AR, United States

**Keywords:** functional plasticity, video gaming, brain development, resting state, fMRI

## Abstract

This study examines whether a decrease in brain development is observable after players have reduced their video gaming time over a period of 1 year. Both video gaming experts and non-experts were recruited, whose resting-state functional MRI (fMRI) data were collected at the beginning and the end of the study. Immediately after the first scan, the participants were instructed to spend no more than 3 h on video gaming weekly for 1 year. The results showed decreased self-reported video gaming skills and decreased amplitude of low-frequency fluctuation (ALFF) in the experts at the end of the study, demonstrating that a reduction in video gaming time over a period of 1 year produced a decrease in brain development. The non-experts served as a control group and had no significant changes. The findings support the adaptive effect of video gaming experience on brain and cognitive development.

## Introduction

Technology has changed the way we live. One of the most prominent changes to the contemporary lifestyle is the use of computers, which are important tools of communication, creativity, and even entertainment. Indeed, video games are becoming increasingly popular across a wide age range worldwide, which was demonstrated by the report that there were approximately 2.2 billion people playing video games worldwide, who spent 3 billion hours in total on video gaming weekly (Kühn et al., 2013). Thus, video games are becoming a major channel through which we learn, understand, and interact with the environment.

The earliest form of video games was typically a single-player *action* video game (AVG; e.g., Super Mario Bros, Tetris, and Unreal Tournament 2004), which prioritized the action component in sensorimotor tasks such as avoiding obstacles by pressing keys or aiming and shooting at targets using a mouse. With the development of electronic and internet technology, video gaming can now be a form of online, organized, multiplayer competition often referred to as electronic sports, which requires not only sensorimotor skills but also strategizing and cooperating with teammates similar to conventional sports such as football, basketball, and soccer (Gong et al., 2016). The interaction between humans in the virtual environment of video games can be similar to that in the real-world environment, as they both require primary and higher-level brain functions such as sensorimotor control, attention, memory, and socioemotional communication (Latham et al., 2013b). Thus, video gaming offers us a new opportunity to understand how learning induces brain plasticity—a major, unresolved question in neuroscience (Bediou et al., 2018).

The effect of video gaming on brain plasticity has attracted increasing research attention over the past few decades. Most of the research has focused on the influence of the *action component* of video gaming on brain development. For example, behavioral findings showed that action video gaming experience was related to improvements in attention (Green and Bavelier, 2003, 2006; Chisholm et al., 2010; Mishra et al., 2011), hand-eye coordination (Jones et al., 1976), response efficiency (Kennedy et al., 2011; West et al., 2013), visual perception (Li et al., 2009; Colzato et al., 2012), and working memory (Boot et al., 2008; Colzato et al., 2012; Blacker and Curby, 2013), while neuroimaging research suggested that the action component of video gaming correlated with increased gray matter volume in the dorsal striatum (Erickson et al., 2010), right posterior parietal cortex (Tanaka et al., 2013), hippocampus, occipital cortex, and right dorsolateral prefrontal cortex (Kühn and Gallinat, 2013; Kühn et al., 2013), suggesting the cognitive and neural benefit of video gaming experience.

Recently, Gong and colleagues examined the effect of League of Legends (LOLs) on brain development (Gong et al., 2015, 2016, 2017; Qiu et al., 2018). LOL is a typical, popular form of e-sports, taking the form of online, organized, multiplayer competitions. A survey conducted in 2014 showed that LOL was played by over 67 million people per month, 27 million people per day, and over 7.5 million people concurrently during peak hours^1^. Cross-sectional, comparative studies on LOL experts and non-experts found that LOL experience was related to improvements in functional integration, structural connections, and topological characteristics of brain networks, including the central executive network (CEN), attentional network, and sensorimotor network (Gong et al., 2015, 2016, 2017; Qiu et al., 2018). A recent interventional study found cognitive and neural plasticity after a 1-h video gaming session in both LOL experts and non-experts under experimental conditions (Qiu et al., 2018), suggesting that the video gaming experience can produce rapid improvements in brain development.

These findings have significantly improved our understanding of brain development related to learning in a virtual environment. However, the effect of video gaming on brain development still needs further exploration. First, as Bediou et al. (2018) suggested, little research has tracked the effect of video gaming experience on brain development *over a period longer than 50 h* (Lledo et al., 2006). This may be due to the logistic difficulty of retaining participants throughout a longitudinal training study, which is usually much shorter than the acquisition of expertise in the real world, which can take several years to complete. This can pose a challenge to research on brain plasticity, as brain development usually occurs through long-term, continuous training. Thus, the retention of the adaptive effect of video gaming on brain development still remains unclear. Second, experimental research has usually exposed participants to a video gaming session and examined how an increase in video gaming time improved brain development (Mishra et al., 2011; Wu et al., 2012; Wang et al., 2017). However, to the best of our knowledge, research has not yet examined whether a decrease in brain development is observable after players have reduced their video gaming time over an extended period of time. Just as astronomers are not satisfied to study only the light side of the moon, researchers in brain development recognize that the dark side—the decrease in brain improvement—holds secrets to a process that must be unlocked.

The current study examined whether a decrease in brain development can be observed after players have reduced their video gaming time over a period of 1 year. This study recruited both LOL experts and non-experts whose resting-state functional MRI (fMRI) data were collected at the beginning and the end of the study. Immediately after the first scan was obtained at the beginning of the study, both the experts and non-experts were instructed to spend no more than 3 h on video gaming *weekly* for 1 year. After this 1-year period, the participants’ resting-state fMRI were obtained again at the end of the study. Participants’ first and second fMRI data were compared to evaluate the within- and between-group differences. Specifically, this study addressed whether a reduction in video gaming time over a period of 1 year could produce a decrease in brain development. Based on the logic that video gaming experience is related to brain plasticity, we predicted that a decrease in brain development should be observed in the second set of fMRI data. The results are essential in understanding the retention of the adaptive effect of extensive learning, which is central to any complete theory of brain plasticity.

This study examined the changes in brain function by analyzing the amplitude of low-frequency fluctuation (ALFF), which was calculated through the square root of the power spectrum within a specific frequency range (usually 0.01–0.08 Hz). ALFF is a widely used method for assessing spontaneous fluctuations in fMRI signal intensity for a given region under the resting state (Zang et al., 2007). Previous research has demonstrated that ALFF is a highly reliable and reproducible method to detect the changes of brain function at the voxel level (Zhang et al., 2010; Zuo et al., 2010; Wang et al., 2011; Guerra-Carrillo et al., 2014; Zuo and Xing, 2014).

## Materials and Methods

### Participants

All participants were right-handed as confirmed by the Edinburgh Handedness Questionnaire (Oldfield, 1971), had normal or corrected-to-normal vision and normal hearing, and did not have a history of neurological or psychiatric illnesses. All participants accepted the protocol, which was approved by the ethics research committee of the University of Electronic Science and Technology of China (UESTC). The study complied with the ethical standards outlined by the Declaration of Helsinki. This study was approved by the research ethics committee of the UESTC and was performed in accordance with the Code of Ethics of the World Medical Association (Declaration of Helsinki). The consent obtained from all participants was both informed and written.

Prior to this study, a survey was given to a large group of individuals who were asked to report their LOL gaming experience (in years) and their Expertise Ranking provided by the LOL software. Only the individuals who were identified as either experts or non-experts were invited to participate in this study. The recruitment of both LOL experts and non-experts enables us to address three issues. First, the effect of long-term video gaming experience and brain plasticity can be addressed through between-group analyses of the data collected at the beginning of the study. Second, the effect of a reduction of video gaming time on brain development can be evaluated through within-group comparisons of the experts’ data collected at the beginning and the end of the study. In addition, between-group comparisons of the fMRI data at the end of the study allow us to evaluate whether the experts’ brain development is comparable to the non-experts after a reduction of video gaming time over a period of 1 year. Third, the non-expert group serves as a control group, allowing us to examine whether a decrease in brain development can be observed under the same experimental instruction yet with a largely unchanged video gaming habit. In addition, the potential reduction in brain development in the experts at the end of study (if any) may be either due to the reduction in their video gaming time or merely driven by maturation. Thus, the non-expert control group also enables us to evaluate whether a reduction in brain development is observable simply across a period of 1 year.

It should be noted that the research question can also be addressed using another experimental design, where LOL experts are randomly assigned into one of the two conditions, in which they are instructed to either maintain their gaming habit or reduce their gaming time. We did not use this alternative experimental design because: (i) this design does not allow us to examine the effect of long-term gaming experience on brain development; (ii) this design does not enable us to determine whether the experts’ brain development is comparable to the non-experts at the end of the study; and (iii) this study is part of a campus-wide project on the effect of reducing students’ gaming time and their cognitive and socio-emotional development. Thus, maintaining experts’ gaming time runs counter to the overall mission of the campus-wide project assigned by the ethics research committee of the UESTC.

The 40 participants were healthy male undergraduate students from the UESTC, including 20 LOL experts (*M* = 21.42 ± 1.64 years of age) and 20 non-experts (*M* = 22.25 ± 1.65 years of age). Five additional participants were excluded from the final sample because of failure to complete the study (*n* = 3) and poor quality fMRI data (*n* = 2).

Group membership was defined by both time- and skill-based criteria, which were identical to the criteria used in previous research (Gong et al., 2016, 2019). The expert group had at least 3 years of LOL experience and were recognized as Gold or higher-level LOL players according to their Ladder-Rank score measured on Elo’s chess-skill rating scale—an objective, widely used method for calculating the relative skill levels of LOL players^2^. The experts were among the Diamond I ~ Gold I according to the aforementioned ranking. All of the non-experts had less than 1.5 years of LOL experience and were recognized as Gold V or lower-level LOL players. Individuals who were naïve to LOL were excluded because their LOL gaming time was (or was close to) zero and therefore, hardly reducible.

Prior to the first MRI scan, participants completed: (i) a questionnaire reporting their age, length of education, and residential status; (ii) an IQ assessment using the Raven Matrices Test; and (iii) a self-report of their LOL ranking expertise ranging from Bronze V to Diamond I—an assessment tool used by the gaming software as well (see Table 1 for results). Every effort was taken to match the two groups in their age, length of education, and residential status (i.e., all participants lived in the on-campus dormitory). Immediately after the first resting-state fMRI scan, both groups received the same instruction of “spending no more than 3 h on video gaming per week throughout a period of 1 year starting from today.” Thus, under the same instruction, a reduction of weekly video gaming time was implemented in the experts, but video gaming time remained largely unchanged in the non-experts since their weekly video gaming time was 2.95 h (SD = 2.27; Table 1). The same instruction was used for both groups to minimize the possible influence of the participants’ awareness of the purpose of the study on their behaviors. Furthermore, participants were contacted regularly to ensure that the reduction in video gaming was implemented. At the end of the study (1 year later), participants’ resting-state fMRI data were collected again. In addition, they were asked to self-report their change in video gaming capability by comparing it against the original video gaming capability self-reported 1 year ago.

**Table 1:** Demographic and behavioral results.

Item	Experts	Non-experts	Statistics
Age	21.42 ± 1.64	22.25 ± 1.65	*t*_(38)_ = 1.57, *p* = 0.13
Video gaming time before the first scan (hours/week)	11.18 ± 7.31	2.95 ± 2.27	*t*_(38)_ = 4.77, *p* < 0.001
Gaming skill level before the first scan (5-point: 1 = Bronze, 5 = Diamond)	4.05 ± 0.78	2.78 ± 0.88	*t*_(38)_ = 4.68, *p* < 0.001
The change of gaming skill level self-reported at the end of the study (5-point: −2 = decreased a lot, 2 = increased a lot)	−1.1 ± 0.55	−0.25 ± 0.91	*t*_(38)_ = 3.56, *p* = 0.001
Length of school education	15 years	15 years	-
Raven’s Progressive Matrices score (percentile)	91.31 ± 5.2	92.77 ± 6.2	*t*_(38)_ = 0.77, *p* = 0.44

### Scanning Procedure

Functional and structural images were acquired using a 3T MRI scanner (GE Discovery MR750) at the MRI research center of UESTC. Resting-state fMRI data were acquired using gradient-echo EPI sequences [repetition time (TR) = 2,000 ms, echo time (TE) = 30 ms, flip angle (FA) = 90°, matrix = 64 × 64, 3 × 3 × 3 mm voxels, field of view (FOV) = 24 × 24 cm^2^, slice thickness/gap = 4 ms/0.4 mm] and an eight channel-phased array head coil. All participants underwent a 510-s resting-state scan to generate 255 volumes (32 slices per volume). High-resolution T1-weighted images were acquired using a three-dimensional fast spoiled gradient echo (T1-3D FSPGR) sequence [TR = 6.008 ms, TE = 1.984 ms, FA = 90°, matrix = 256 × 256 mm voxels, FOV = 25.6 × 20 cm^2^ (80%), slice thickness (no gap) = 1 mm] to generate 152 slices.

### Resting State Functional MRI Data Preprocessing

The fMRI data were first preprocessed according to typical preprocessing procedures using SPM8 (Wellcome Department of Cognitive Neurology, London, UK), including removal of the first five volumes of each run, slice scan time correction, head motion correction, and normalization of the images using an EPI template in the Montreal Neurological Institute (MNI) atlas space. Spatial smoothing was applied using a Gaussian kernel of 8 mm full-width at half-maximum (FWHM). Temporal filtering (bandpass) was between 0.01 and 0.08 Hz, and the global mean signal of the whole brain was removed.

### Computation of the Amplitude of Low-Frequency Fluctuation (ALFF) Map

After preprocessing, the resting-state fMRI data were utilized to compute the ALFF map using DPABI (Yan et al., 2016). The ALFF map was built according to the standard procedures established by previous research (Guerra-Carrillo et al., 2014). The filtered time series was transformed to the frequency domain using the fast Fourier transform (FFT). Since the power of a given frequency was proportional to the square of the amplitude of this frequency component, the square root was calculated at each frequency of the power spectrum, and the averaged square root was obtained across 0.01–0.08 Hz at each voxel. This averaged square root was taken as the ALFF. For standardization, the ALFF of each voxel was divided by the global mean ALFF value for each participant, resulting in a relative ALFF (Cantou et al., 2018).

Past research has demonstrated that ALFF results can reliably indicate brain activities in a resting brain (Xing and Zuo, 2018; Zuo et al., 2019). In addition, in order to verify the ALFF results, this study also analyzed the local functional connectivity density (local FCD), which is a data-driven measure for local changes of brain functions at voxel level just like ALFF. However, it should be noted that despite the strong association between the ALFF and local FCD data (Tomasi et al., 2016), they are two different measures with the local FCD indicating the local functional integration (Tomasi and Volkow, 2010) while the local power spectrum (ALFF) reflecting the local power spectrum.

### Statistical Analysis

Between-group comparisons of demographic and behavioral data were conducted through independent samples *t*-tests. Imaging data were analyzed through *t*-tests and a repeated measure ANOVA using SPM8 software. For the ALFF analysis, one-sample *t*-tests were performed for the four conditions (e.g., experts’ first scan, experts’ second scan, non-experts’ first scan, non-experts’ second scan). Then, 2 (group: experts vs. non-experts) × 2 (time: first scan vs. second scanning) ANOVA analyses were performed. The multiple comparisons were corrected using a false discovery rate (FDR) of *p* < 0.05 throughout this study. Maps are projected on a 3D brain surface with the BrainNet Viewer (Xia et al., 2013).

## Results

### Demographic and Behavioral Results

Between-group comparisons were conducted through separate independent-samples *t*-tests. The results showed that the two groups did not differ in their age, IQ, or length of education (Table 1). In addition, all participants had the same residential status, living in on-campus dormitory. This ensured that they had similar daily schedules, thus minimizing the possible influence of confounding factors unrelated to video gaming experience on human development. Consistent with the Ladder-Rank score provided by the LOL software, the experts also self-reported a significantly higher level of video gaming capability than the non-experts did at the beginning of the study, thus verifying the definition of group membership in this study. However, the experts self-reported a significantly greater decrease in video gaming capability than the non-experts did at the end of the study (Table 1).

### ALFF Results

**Figure 1:**
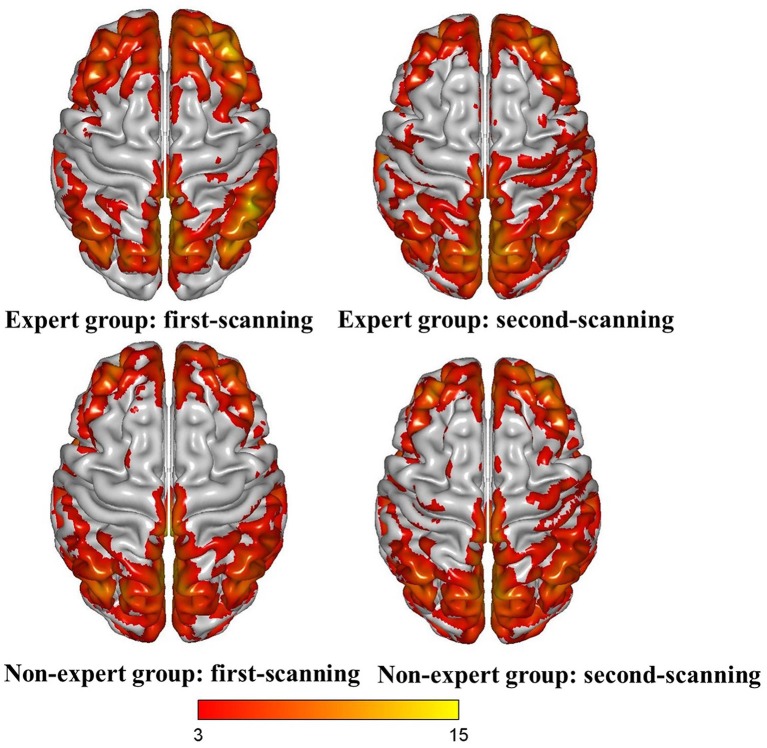
The amplitude of low-frequency fluctuation (ALFF) maps of one-sample *t*-test [*p* < 0.05, false discovery rate (FDR)-corrected, cluster size > 50].

**Figure 2:**
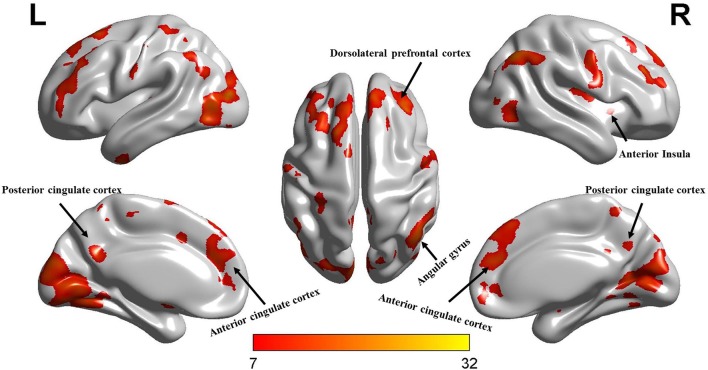
The ALFF maps of interaction effects between the group and time factors. Colors from yellow to red indicate an increasing *F*-value (*p* < 0.05, FDR-corrected, cluster size > 50).

Separate one-sample *t*-tests were conducted for the four conditions (i.e., experts’ first scan, experts’ second scan, non-experts’ first scan, and non-experts’ second scan). The results showed a similar spatial distribution across the four conditions (Figure 1). Then, a 2 (group: experts vs. non-experts) × 2 (time: first scan vs. second scan) ANOVA was conducted. Main effects of group and time emerged, but these main effects were qualified by a group × time interaction. Further analyses were conducted to deconstruct the interaction. The ALFF maps of the group × time interaction revealed the main nodes of three intrinsic networks, including the default mode network (DMN), CEN, and salience network (SN). Specifically, the maps included: (i) the posterior cingulate cortex (PCC) and right angular gyrus of the DMN; (ii) the right dorsolateral prefrontal cortex of the CEN; and (iii) the anterior cingulate cortex (ACC) and right anterior insula of the SN (Figure 2).

**Figure 3:**
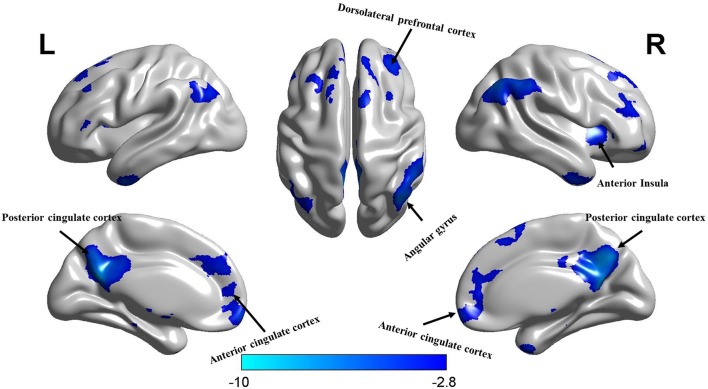
The ALFF maps of comparisons between the first and the second scanning in the experts. Colors from blue to azure indicate a decreasing *t*-value (*p* < 0.05, FDR-corrected, cluster size > 50).

**Figure 4:**
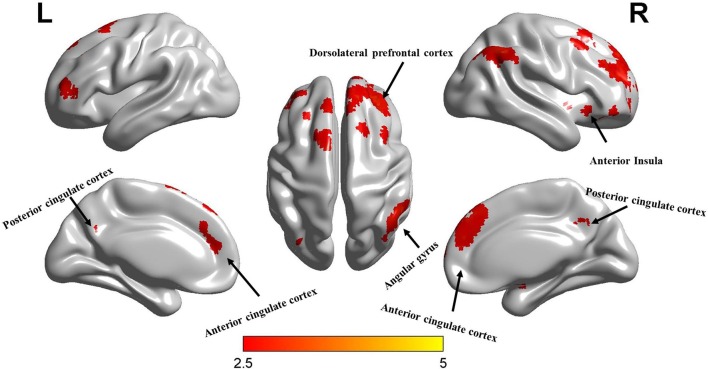
The ALFF maps of comparison between the expert and control group in the first scanning. Colors from red to yellow indicate a significant increasing *t*-value in the expert group over the controls (*p* < 0.05, FDR-corrected, cluster size > 50).

To further deconstruct the interactions, simple-effect analyses were conducted. Specifically, paired-sample *t*-tests on the experts’ first and second scanning data showed significantly decreased ALFF in the second scan (Figure 3). The same analyses were performed for the non-experts, which, however, did not reveal significant differences between the first and second scan. Independent-samples *t*-tests analyzed between-group differences at the beginning and end of the study. The analysis of the first scan data showed that the experts had significantly higher ALFF compared to the non-experts at the beginning of the study (Figure 4). However, the analysis of the second scan data showed no between-group differences at the end of the study. See Supplementary Tables S1–S3 for further results of significant maps. Furthermore, the result of Local FCD verified the result of ALFF (see Supplementary Figure S1 and Supplementary Table S4).

## Discussion

This study examined the influence of video gaming experience on brain development by exploring whether a reduction in gaming time could produce a decrease in brain development. Both LOL experts and non-experts were recruited. The two groups were matched in age, length of education, and residential status. Immediately after the first resting state fMRI scan, both groups were instructed to spend no more than 3 h weekly on video gaming for 1 year. After this 1-year period, the participants’ resting-state fMRI was scanned again at the end of the study. In addition, the participants were asked to self-report their video gaming skills at the beginning of the study and the change of their gaming skills at the end of the study. Three major findings emerged. First, within-group comparisons showed significantly decreased ALFF and self-reported gaming skills (Table 1) in the experts at the end of the study (Figures 2, 3), demonstrating that a decrease in brain development and video gaming skills can be observed in the experts after they reduced their video gaming time over a period of 1 year. However, the within-group analysis for the non-experts did not find a decrease in brain development at the end of the study, suggesting that this decreased brain development was more readily observable in experts than non-experts. Second, between-group comparisons showed that experts had a significantly higher level of ALFF and self-reported video gaming skills at the beginning of the study, revealing the effect of long-term video gaming experience on brain and cognitive development. Furthermore, the findings verified the group membership definition used in this study. Third, between-group comparisons showed that the experts and non-experts did not differ in their ALFF at the end of the study, suggesting that the experts’ brain activity was similar to the non-experts after the reduction in video gaming time was implemented for 1 year. Thus, this study showed that a decrease in brain development and video gaming skills was observed after players reduced their video gaming time over a period of 1 year.

For the ALFF analysis, we first conducted one-sample *t*-tests for the four conditions (i.e., experts’ first scan, experts’ second scan, non-experts’ first scan, and non-experts’ second scan). Consistent with previous research (Han et al., 2011), a similar pattern of spatial distribution was observed across the four conditions (Figure 1). We then performed a 2 (group) × 2 (time) ANOVA and found significant main effects of group and time and a group × time interaction (Figure 2). Given that the non-experts’ ALFF did not significantly change between the two scanning sessions, this interaction should be driven by the experts’ decreased ALFF across the two scanning sessions (see Figure 3).

During a typical LOL gaming session, players usually need to remember multiple units and battlefield landforms (requiring perception and long-term memory), constantly make and update tactical plans under time pressure (requiring attention and working memory), and maneuver units by executing over 200 bimanual actions per minute by keyboard and mouse (requiring attention and sensorimotor integration; Latham et al., 2013a; Gong et al., 2015, 2016). Therefore, frequent playing may enhance these cognitive functions, as suggested by previous studies [e.g., visual processing (Green and Bavelier, 2007; Li et al., 2009, 2011), including eye-hand coordination (Jones et al., 1976), contrast sensitivity (Li et al., 2009), oculomotor performance (West et al., 2013), body movement (Kennedy et al., 2011), selective attention (Green and Bavelier, 2003), spatial distribution of visuospatial attention (Green and Bavelier, 2006), attentional capture (Chisholm et al., 2010), attention shifting (Cain et al., 2012), cognitive control (Colzato et al., 2012), and visual short-term and working memory (Colzato et al., 2012; Blacker and Curby, 2013; Blacker et al., 2014)]. Consistent with the literature, this study revealed significantly higher ALFF in the experts compared with the non-experts *at the beginning of the study*, including differences in the PCC and right angular gyrus of the DMN, the right dorsolateral prefrontal cortex of the CEN and the ACC and right anterior insula of the SN (see Figure 2). Based on the same logic, we predicted that a reduction in video gaming time should produce decreases in ALFF and the relevant brain areas. The current findings in the experts confirmed this prediction, thus supporting the effect of video gaming experience on brain plasticity.

ALFF is an index at the voxel level for detecting the intensity of spontaneous fluctuations in BOLD signal and may represent a baseline of brain activity for the resting state (Han et al., 2011). Research has shown that ALFF is an effective, reliable indicator of brain plasticity (Zhang et al., 2010; Han et al., 2011). Furthermore, the DMN can be activated when the task requires players to think about others and themselves and remembering the past and planning for the future (Anticevic et al., 2012; Raichle, 2015). In addition, the SN supports the detection of salient events, while the CEN supports attentional control and working memory (Cocchi et al., 2013). Although the literature has suggested that the DMN often competes against the CEN (Anticevic et al., 2012; Cocchi et al., 2013; Raichle, 2015) for cognitive resources, there is also evidence showing that the activation of the DMN may facilitate the performance of the CEN in the tasks where the function of the CEN is highly dependent on the information processed by the DMN (e.g., the information stored in the long-term memory; Spreng et al., 2014). Thus, during a typical LOL session, the activation of the DMN may assist the CEN in the retrieval of relevant information from the long-term memory, which enhances players’ performance.

Furthermore, we found that the experts’ and non-experts’ ALFF did not differ at the end of the study. To the best of our knowledge, this study is the first to show that video gaming expertise, which is acquired through several years of gaming experience, may be unobservable after the players reduce their video gaming time over a course of 1 year. However, several issues remain. First, it is unclear whether this finding suggests a disappearance of video gaming expertise in the experts at the end of the study. Perhaps the experts’ video gaming expertise can be observed in other tasks or using other neuroscience methods. Second, can the experts regain their video gaming expertise? If so, can their regaining of expertise happen faster than a novice acquires video gaming expertise? These issues are essential for a complete theory of the neural basis of learning and will be addressed by future research. Finally, it should be noted that the current research question may also be addressed using another experimental design where two groups of *experts* are recruited, one of whom is instructed to reduce their video gaming time while the other is instructed to remain or even increase their video gaming time. Although under this design, experimental instructions will differ between groups, future research should also use this experimental design to offer another perspective on the effect of a reduction in video gaming on brain development.

Nevertheless, it should be noted that manipulating participants’ behavior on a daily basis over a period of 1 year is logistically challenging. Thus, this study used a simplified, implementable instruction that asked the participants to spend no more than 3 h on video gaming for 1 year. Further complicating the experimental procedure is the fact that this study examined the *lack* of training on brain development. A complete, accurate examination of a lack of experience requires a continuous record of participants’ behavior for the entirety of the project, which is beyond the scope of the current study. Thus, this study cannot quantify the degree of the reduction in video gaming time within a participant. As a result, this study does not allow us to evaluate the association between the degree of the reduction in video gaming time and the extent of the ALFF decrease. Thus, it is still unclear whether the degree of the video gaming time reduction can predict the extent of ALFF decrease within a participant. This is a remaining issue that should be addressed by future research. However, to the best of our knowledge, this study is the first to show that a reduction in video gaming time can produce a decrease in brain activity, thus supporting the cognitive and neural effect of video gaming experience.

## Ethics Statement

All participants accepted the protocol, which was approved by the ethics research committee of the University of Electronic Science and Technology of China (UESTC). The study complied with the ethical standards outlined by the Declaration of Helsinki. This study was approved by the research ethics committee of the UESTC and was performed in accordance with the Code of Ethics of the World Medical Association (Declaration of Helsinki). The consent obtained from all participants was both informed and written.

## Author Contributions

DG, WM and DY conceived and designed this study. DG and YY performed the study. YP and XG analyzed the data. DG, WM, YY and DY wrote the main manuscript text. DG and WM prepared the figures and tables. All authors reviewed the manuscript.

## Conflict of Interest Statement

The authors declare that the research was conducted in the absence of any commercial or financial relationships that could be construed as a potential conflict of interest.

## Footnotes

^1^http://blogs.wsj.com/digits/2014/01/27/player-tally-for-league-of-legends-surges/

^2^LOL consists of three game modes: Summoner’s Rift (Ranked Matchmaking), Twisted Treeline, and Howling Abyss (Normal Matchmaking). Players’ gaming experience and expertise are indicated by their Ranked Matchmaking level which is calculated based on the Elo rating system—a method for calculating the relative skill levels of players in competitor-vs.-competitor games. The Elo rating system has multiple stages—Bronze, Silver, Gold, Platinum, Diamond, Master, and Challenger—across which the expertise level increases sequentially. Each stage has five phases ranging from V to I. In the Ranked Matchmaking mode, players who win (or lose) a game will gain (or lose) a certain amount of points depending on the champion’s performance. Winning each 100 points activates the promotion competition, the successful completion of which promotes the player to the next stage or level. The successful maintenance of points requires one to play LOL on a regular and frequent basis.
