# Supplementary material for: A Reduction in Video Gaming Time Produced a Decrease in Brain Activity
Source: Front Hum Neurosci. 2019 Apr 17;13:134. doi: 10.3389/fnhum.2019.00134 (PMC6478706; doi:10.3389/fnhum.2019.00134)
Supplement: Supplementary file 1 [file Table_1.DOCX]

**Supplementary materials**

**Supplementary Table 1.**

The ALFF results of interaction effects between the group and time factors. L=Left, R = Right, Mid = Middle, Sup = Superior, Inf = Inferior, Ant= Anterior, Med = Medial, Orb = orbit, Supp = Supplementary.

| **Clusters** | **Brain regions**  **(AAL template)** | **The number of voxels** | **Peak**  ***F*-value** | **Peak MNI Coordinate**  **[x y z]** |
| --- | --- | --- | --- | --- |
|  | Lingual_L | 354 |  |  |
|  | Calcarine_L | 294 |  |  |
|  | Occipital_Mid_L | 269 |  |  |
|  | Calcarine_R | 241 |  |  |
|  | Lingual_R | 186 |  |  |
|  | Occipital_Sup_L | 160 |  |  |
| 1 | Cuneus_L | 154 | 21.81 | -36 -84 12 |
|  | Fusiform_L | 131 |  |  |
|  | Occipital_Inf_L | 121 |  |  |
|  | Precuneus_L | 93 |  |  |
|  | Cuneus_R | 91 |  |  |
|  | Parahippocampa Gyrus | 48 |  |  |
|  |  |  |  |  |
|  | Temporal_Inf_R | 52 |  |  |
| 2 | Temporal_Mid_R | 49 | 13.95 | 48 -72 -6 |
|  | Occipital_Mid_R | 32 |  |  |
|  |  |  |  |  |
|  | Frontal_Mid_L | 403 |  |  |
|  | Frontal_Sup_Medial_L | 289 |  |  |
|  | Frontal_Sup_Medial_R | 278 |  |  |
|  | Frontal_Sup_L | 267 |  |  |
| 3 | Frontal_Sup_R | 123 | 32.58 | -9 42 57 |
|  | Cingulum_Ant_L | 85 |  |  |
|  | Cingulum_Ant_R | 69 |  |  |
|  | Frontal_Med_Orb_R | 50 |  |  |
|  | Supp_Motor_Area_L | 34 |  |  |
|  |  |  |  |  |
|  | Precentral_R | 101 |  |  |
| 4 | Postcentral_R | 83 | 14.71 | 48 -6 33 |
|  | Rolandic_Oper_R | 63 |  |  |
|  | Insula_R | 40 |  |  |
|  |  |  |  |  |
| 5 | Frontal_Mid_R | 214 | 20.25 | 36 39 33 |
|  |  |  |  |  |
| 6 | Precuneus_L | 59 | 14.28 | -6 -54 27 |
|  | Precuneus_R | 38 |  |  |
|  |  |  |  |  |
| 7 | Angular_L | 60 | 17.47 | -54 -57 48 |
|  |  |  |  |  |
| 8 | Angular_R | 168 | 20.03 | 45 -63 42 |
|  | Parietal_Inf_R | 94 |  |  |
|  |  |  |  |  |
| 9 | Postcentral_L | 72 | 17.09 | -30 -33 60 |

**Supplementary Table 2.**

The ALFF results of comparisons between the first and the second scanning in the experts. L=Left, R = Right, Mid = Middle, Sup = Superior, Inf = Inferior, Ant= Anterior, Med = Medial, Orb = orbit, Supp = Supplementary.

| **Clusters** | **Brain regions**  **(AAL template)** | **The number of voxels** | **Peak**  ***t*-value** | **Peak MNI Coordinate**  **[x y z]** |
| --- | --- | --- | --- | --- |
| 1 | Temporal_Inf_L | 155 |  |  |
|  | Temporal_Inf_R | 153 |  |  |
|  | ParaHippocampal_L | 71 | -9.68 | -9 15 -30 |
|  | Temporal_Pole_Mid_R | 66 |  |  |
|  | Fusiform_L | 55 |  |  |
|  |  |  |  |  |
|  | Frontal_Med_Orb_L | 115 |  |  |
|  | Frontal_Med_Orb_R | 108 |  |  |
|  | Rectus_L | 59 |  |  |
| 2 | Frontal_Sup_Orb_L | 55 | -7.99 | -6 66 -12 |
|  | Cingulum_Ant_L | 51 |  |  |
|  | Rectus_R | 34 |  |  |
|  | Frontal_Sup_Orb_R | 32 |  |  |
|  |  |  |  |  |
| 3 | Insula_R | 66 | -4.83 | 42 12 3 |
| 4 | Insula_L | 28 | -3.58 | -39 12 6 |
|  | Frontal_Inf_Tri_L | 154 |  |  |
|  |  |  |  |  |
|  | Frontal_Mid_L | 83 |  |  |
| 5 | Frontal_Inf_Oper_L | 60 | -6.01 | -57 21 24 |
|  | Precentral_L | 23 |  |  |
|  |  |  |  |  |
|  | Precuneus_R | 437 |  |  |
|  | Precuneus_L | 372 |  |  |
|  | Cingulum_Post_L | 125 |  |  |
| 6 | Cingulum_Post_R | 110 | -8.21 | 6 -54 36 |
|  | Cingulum_Mid_R | 98 |  |  |
|  | Cuneus_L | 84 |  |  |
|  | Cingulum_Mid_L | 83 |  |  |
|  | Calcarine_L | 40 |  |  |
|  |  |  |  |  |
|  | Frontal_Mid_R | 159 |  |  |
| 7 | Frontal_Inf_Tri_R | 67 | -4.12 | 42 48 27 |
|  | Frontal_Inf_Orb_R | 22 |  |  |
|  |  |  |  |  |
|  | Angular_L | 192 |  |  |
| 8 | Occipital_Mid_L | 134 | -5.89 | -51 -75 30 |
|  | Parietal_Inf_L | 66 |  |  |
|  |  |  |  |  |
|  | Angular_R | 216 |  |  |
| 9 | SupraMarginal_R | 93 | -4.97 | 54 -69 33 |
|  | Occipital_Mid_R | 54 |  |  |
|  | Parietal_Inf_R | 49 |  |  |
|  |  |  |  |  |
| 10 | Frontal_Sup_L | 62 | -4.86 | -15 39 54 |
|  | Frontal_Sup_Medial_L | 26 |  |  |
|  |  |  |  |  |
|  |  |  |  |  |

**Supplementary Table 3.**

The ALFF results of comparison between the expert and control group in the first scanning. L=Left, R = Right, Mid = Middle, Sup = Superior, Inf = Inferior, Ant= Anterior, Med = Medial, Orb = orbit, Supp = Supplementary.

| **Clusters** | **Brain regions**  **(AAL template)** | **The number of voxels** | **Peak**  ***t*-value** | **Peak MNI Coordinate**  **[x y z]** |
| --- | --- | --- | --- | --- |
| 1 | Rectus_L | 61 | 3.83 | -3 24 -27 |
| 2 | ParaHippocampal_L | 59 | 3.2 | -21 3 -21 |
|  |  |  |  |  |
|  | Frontal_Mid_L | 569 |  |  |
|  | Frontal_Mid_R | 569 |  |  |
|  | Frontal_Sup_Medial_L | 403 |  |  |
|  | Frontal_Sup_L | 353 |  |  |
|  | Frontal_Sup_Medial_R | 340 |  |  |
|  | Frontal_Sup_R | 335 |  |  |
|  | Frontal_Inf_Orb_R | 142 |  |  |
|  | Frontal_Inf_Tri_L | 116 |  |  |
|  | Frontal_Inf_Tri_R | 105 |  |  |
| 3 | Insula_R | 88 | 6.04 | -9 42 54 |
|  | Supp_Motor_Area_L | 86 |  |  |
|  | Cingulum_Ant_L | 83 |  |  |
|  | Frontal_Med_Orb_L | 68 |  |  |
|  | Temporal_Pole_Sup_R | 65 |  |  |
|  | Frontal_Med_Orb_R | 64 |  |  |
|  | Frontal_Mid_Orb_R | 56 |  |  |
|  | Cingulum_Ant_R | 52 |  |  |
|  | Frontal_Inf_Orb_L | 52 |  |  |
|  | Cingulum_Mid_L | 50 |  |  |
|  | Cingulum_Mid_R | 44 |  |  |
|  |  |  |  |  |
| 4 | Insula_L | 84 | 3.86 | -30 15 12 |
|  |  |  |  |  |
|  | Angular_L | 106 |  |  |
| 5 | Occipital_Mid_L | 71 | 4.43 | -33 -75 33 |
|  | Temporal_Mid_L | 34 |  |  |
|  |  |  |  |  |
| 6 | Precuneus_R | 82 | 4.25 | 15 -45 36 |
|  | Precuneus_L | 72 |  |  |
|  |  |  |  |  |
|  | Angular_R | 164 |  |  |
| 7 | Parietal_Inf_R | 112 | 4.11 | 45 -63 42 |
|  | Parietal_Sup_R | 35 |  |  |
|  | Occipital_Mid_R | 34 |  |  |
|  |  |  |  |  |


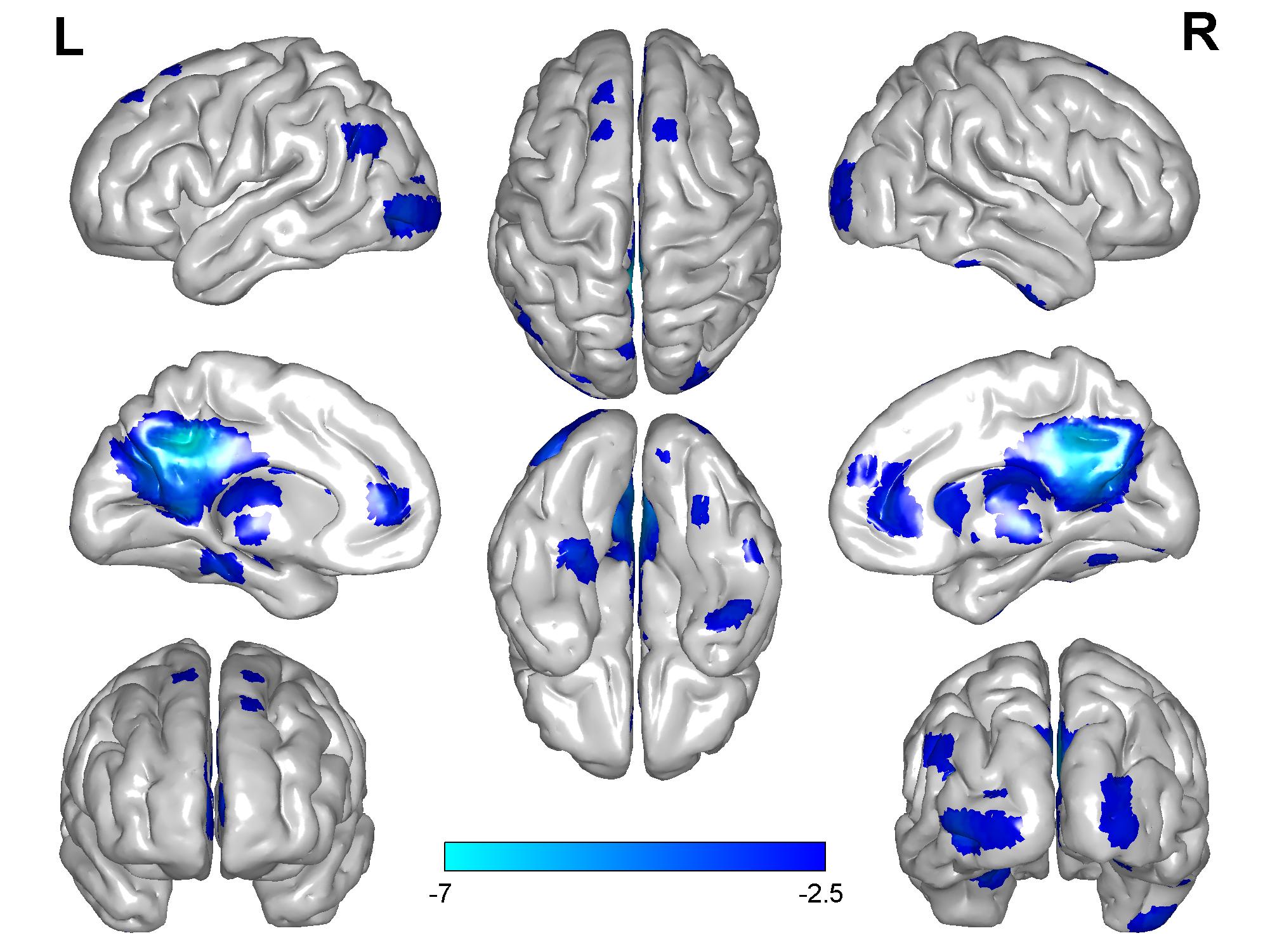


Supplementary Fig.1. The Local FCD maps of comparisons between the first and the second scanning in the experts. Colors from blue to azure indicate a decreasing t-value (p < 0.05, FDR-corrected, cluster size > 50).

**Supplementary Table 4.**

The local FCD results of comparisons between the first and the second scanning in the experts. L=Left, R = Right, Mid = Middle, Sup = Superior, Inf = Inferior, Ant= Anterior, Med = Medial, Orb = orbit, Supp = Supplementary.

| **Clusters** | **Brain regions**  **(AAL template)** | **The number of voxels** | **Peak**  ***t*-value** | **Peak MNI Coordinate**  **[x y z]** |
| --- | --- | --- | --- | --- |
| 1 | Precuneus_R | 361 |  |  |
|  | Precuneus_L | 315 |  |  |
|  | Medial Frontal Gyrus | 110 |  |  |
|  | Cingulum_Post_L | 109 |  |  |
|  | Cingulum_Mid_R | 93 |  |  |
|  | Cuneus_L | 93 |  |  |
|  | Cingulum_Ant_R | 91 | -7.13 | -6 -48 36 |
|  | Cingulum_Mid_L | 91 |  |  |
|  | Cingulum_Post_R | 90 |  |  |
|  | Calcarine_L | 73 |  |  |
|  | Angular_L | 65 |  |  |
|  | Middle Temporal Gyrus | 57 |  |  |
|  | Cingulum_Ant_L | 45 |  |  |
|  | Calcarine_R | 38 |  |  |
|  |  |  |  |  |
| 2 | Occipital_Mid_R | 85 | -3.91 | 27 -81 9 |
|  |  |  |  |  |
| 3 | Occipital_Mid_L | 39 | -3.75 | -39 -81 -3 |
|  | Occipital_Inf_L | 31 |  |  |
|  |  |  |  |  |
| 4 | Fusiform_L | 22 | -4.21 | 12 -63 -21 |
|  |  |  |  |  |
